# Phytochemical Analysis and Anti‐Lipid Accumulation Effects of Pulsed Electric Field (PEF)‐Processed Black Rice and Green Tea Extracts in Oleic Acid‐Induced Hepatocytes

**DOI:** 10.1002/fsn3.70329

**Published:** 2025-05-23

**Authors:** Narisara Paradee, Thararat Yimcharoen, Niramon Utama‐ang, Kornvipa Settakorn, Hataichanok Chuljerm, Somdet Srichairatanakool, Pimpisid Koonyosying

**Affiliations:** ^1^ Department of Biochemistry, Faculty of Medicine Chiang Mai University Chiang Mai Thailand; ^2^ Cluster of High Value Products From Thai Rice and Plants for Health Chiang Mai University Chiang Mai Thailand; ^3^ Clinical Research Center for Food and Herbal Product Trials and Development (CR‐FAH), Faculty of Medicine Chiang Mai University Chiang Mai Thailand; ^4^ Department of Pharmacology, Faculty of Medicine Chiang Mai University Chiang Mai Thailand; ^5^ School of Health Sciences Research, Research Institute for Health Sciences Chiang Mai University Chiang Mai Thailand; ^6^ Environmental‐Occupational Health Sciences and Non Communicable Diseases Research Center Research Institute for Health Sciences Chiang Mai University Chiang Mai Thailand

**Keywords:** black rice, green tea, hepatic steatosis, lipid accumulation, pulsed electric field

## Abstract

High fat consumption leads to the development of fatty liver or hepatic steatosis, which contributes to liver fibrosis, liver cirrhosis, and hepatocellular carcinoma. Early intervention is crucial to prevent this health issue. Black rice ( *Oryza sativa L. indica* ) and green tea ( *Camellia sinensis var. assamica* ) are traditional Thai supplements known for their antioxidant and anti‐inflammatory properties. This study aimed to evaluate the phytochemical composition, biological activities, and anti‐lipid accumulation effect of Pulsed Electric Field (PEF)‐Processed black rice extract (BRE) and green tea extract (GTE) in oleic acid‐induced HepG2 cells. The PEF technology was utilized for extraction, increasing the yield of active compounds. HPLC profiles revealed that BRE is mainly composed of anthocyanins, particularly Cyanidin‐3‐O‐glucoside (C3G), while GTE is rich in catechins, especially epigallocatechin gallate (EGCG). Both extracts showed a strong radical scavenging activity and effective reducing power. In oleic acid‐induced HepG2 cells, monotherapy and combination therapy of the extracts significantly reduced lipid accumulation, AST activity, and IL‐6 levels. These findings suggest that BRE, GTE, and their combination exhibit potent hepatoprotective properties through reducing lipid accumulation, liver damage, and inflammation, highlighting their potential therapeutic application in the management of hepatic steatosis.

## Graphical abstract

Pulsed electric field (PEF)‐processed black rice (BRE) mainly compose Cyanidin‐3‐O‐glucoside, while PEF‐Processed Green Tea Extracts (GTE) is rich in epigallocatechin gallate. BRE and GTE presented powerful radical scavenging and reducing power activities. BRE, GTE and the combination exhibited potent anti‐inflammation and hepatoprotective property. Mono‐ and combination therapy significantly reduced lipid accumulation in lipid‐induced model.

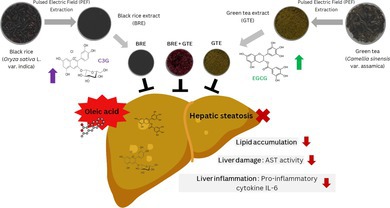

## 1 Introduction

Hepatic steatosis, commonly known as fatty liver disease, is characterized by an imbalance in lipid metabolism leading to the excessive accumulation of lipids within liver cells (Nassir et al. 2015; Santos‐Laso et al. 2021). This condition is a significant public health issue worldwide and is typically associated with dietary and lifestyle factors such as increased calorie intake, high consumption of saturated fats and sugars, and insulin resistance (Marzuillo et al. 2015). Fatty liver diseases occur when fat accumulates in liver cells, leading to steatosis. If this condition persists, it can progress to steatohepatitis, which involves liver cell damage and inflammation due to oxidative stress (Benedict and Zhang 2017). Continued inflammation can result in fibrosis, where excess connective tissue forms. Advanced fibrosis can develop into cirrhosis, characterized by significant scar tissue that alters liver structure and function. This advanced stage, driven by chronic inflammation and tissue scarring, can cause serious health issues like jaundice, high blood pressure in the liver, and an increased risk of liver cancer (HCC) (Reccia et al. 2017). Early intervention, including improving obesity and reducing liver lipid accumulation, is crucial to prevent severe and irreversible liver damage (Huang et al. 2018).

Currently, natural plant ingredients are being utilized to treat various illnesses. Among them, black rice and green tea have gained popularity for their potential health benefits. Black rice ( *Oryza sativa* L. var. indica) has been extensively cultivated and consumed in Southeast Asian countries, including Thailand. Purple rice, recognized by its pericarp color ranging from deep purple to black, is characterized by its high content of phenolic acids and flavonoids, especially anthocyanins (Tyagi et al. 2022). It has been widely reported that anthocyanins exhibit strong antioxidant, anti‐inflammatory, anti‐cancer, and hypoglycemic activities (Hui et al. 2010; Min et al. 2010). Similarly, green tea ( *Camellia sinensis var. assamica* ) is widely grown in Southeast Asian countries, including Thailand, and consumed as a popular beverage for its refreshing taste and potential health benefits. Green tea is rich in polyphenols, particularly catechins, which are potent antioxidants known to have various health‐promoting properties, including antioxidant, anti‐inflammatory, and hepatoprotective effects (Koonyosying et al. 2018; Mota et al. 2015; Ohishi et al. 2016).

In Thailand, several medicinal foods and herbs, such as black rice and green tea, have been recognized for their health benefits. Although many previous studies have demonstrated the hepatoprotective activity of black rice and green tea, only a few have reported on their anti‐lipid effects and cellular mechanisms of action. Therefore, this study aims to investigate phytochemical active compounds and the hepatoprotective effects of black rice extract (BRE), green tea extract (GTE), and their combination through evaluating their anti‐lipid accumulation, anti‐liver damage, and anti‐inflammatory activities in oleic acid‐induced HepG2 cells.

## 2 Materials and Methods

### 2.1 Chemicals and Reagents

All HPLC standards and Trolox were purchased from Sigma‐Aldrich. Acetonitrile, methanol, isopropanol, and Folin–Ciocalteu reagent were obtained from Merck. DPPH radical, ABTS radical, and 2,4,6‐tripyridyl‐s‐triazine (TPTZ) were purchased from Sigma‐Aldrich. Dulbecco's modified Eagle's medium (DMEM) and fetal bovine serum (FBS) were obtained from Gibco. Penicillin–streptomycin, trypsin, and MTT dye were purchased from Invitrogen. AdipoRed Adipogenesis Assay Reagent was obtained from Lonza. Human IL‐6 ELISA kit was purchased from BioLegend.

### 2.2 Preparation of Black Rice and Green Tea Extract

Black rice *(Oryza sativa L. indica)* harvested from fields in Chiang Mai, Thailand, was initially soaked in water for 10 min. Subsequently, the soaked black rice mixture underwent extraction using a pulsed electric field (PEF). Following extraction, the BRE was subjected to continuous shaking using an electric shaker for 6 h. The resulting mixture was then filtered and concentrated to dryness using rotary evaporation to obtain the BRE. BRE powder was stored at −20°C for further analysis.

Green tea ( *Camellia sinensis var. assamica* ) collected from fields in Chiang Mai, Thailand, was promptly dried under vacuum. Following drying, the green tea was ground and subjected to extraction with water using a PEF. The GTE was subsequently filtered and concentrated using rotary evaporation. The resulting GTE dried powder was preserved at −20°C until further analysis.

### 2.3 Characterization of Anthocyanins

Anthocyanins in BRE or GTE were analyzed using a High‐Performance Liquid Chromatography (HPLC) system (SHIMADZU, Japan) equipped with an Allure C18 (25 cm × 4.6 mm, 5 μm) (Ryu et al. 1998). The extracts were reconstituted in 0.5% trifluoroacetic acid (TFA) in 95% ethanol and shaken for 9 h. The samples were then filtered through a nylon membrane (0.45 μm pore size) and subjected to chromatography analysis. Separation was performed using a linear gradient system from 0.1% TFA in H_2_O (mobile phase A) to 0.1% TFA in methanol (mobile phase B) for 30 min. The flow rate was set at 1.0 mL/min, and the column temperature was maintained at 30°C. A UV–Vis diode array detector was set to 280 nm. Anthocyanins were identified by comparing their retention times and peak areas to those of standard compounds, including cyanidin‐3,5‐diglucoside (cyanin chloride), cyanidin‐3‐O‐glucoside chloride (C‐3‐G), cyanidin‐3‐O‐rutinoside chloride, and peonidin‐3‐O‐glucoside chloride (P‐3‐G).

### 2.4 Characterization of Catechins, Caffeine, and Gallic Acid

Catechins in BRE or GTE were analyzed using a HPLC system (SHIMADZU, Japan) equipped with a Platinum EPS C18 100A 3u (53 mm × 7 mm) (Minamisawa et al. 2004; Wang et al. 2000). The extracts were reconstituted in 100% ethanol, filtered through a nylon membrane (0.45 μm pore size) and then subjected to chromatography analysis. Separation was carried out using an isocratic elution system with a mobile phase comprising 0.05% TFA in 13% acetonitrile. The flow rate was set at 1.0 mL/min, and the column temperature was maintained at 30°C. A UV–Vis diode array detector was set to 210 nm. Compounds were identified by comparing their retention times and peak areas to those of standard compounds, including caffeine, gallic acid, catechin (C), epicatechin (EC), epicatechin gallate (ECG) and epigallocatechin gallate (EGCG).

### 2.5 Characterization of Tocopherol

Tocopherols in BRE or GTE were analyzed using a HPLC system (SHIMADZU, Japan) equipped with a C18 reverse‐phase column (250 mm × 4.6 mm, 5 μm) (Pestana‐Bauer et al. 2012). The samples were reconstituted in isopropanol and filtered through a nylon membrane (0.45 μm pore size). The mobile phases were composed of acetonitrile/methanol/isopropanol (v/v/v) in the ratios of 50:40:10 (mobile phase A) and 30:65:5 (mobile phase B). Separation was performed using isocratic elution with mobile phase A for 5 min, followed by a 10‐min gradient transition from mobile phase A to 100% mobile phase B, and a final 5‐min isocratic elution with mobile phase B. The flow rate was set at 1.0 mL/min, and the column temperature was maintained at 30°C. A fluorescence detector was set with excitation and emission wavelengths at 295 nm and 330 nm. Tocopherol quantification was carried out by comparing retention times and peak areas with those of tocopherol standards.

### 2.6 Quantification of Total Phenolic Content

Total phenolic content (TPC) was evaluated using the Folin–Ciocalteu colorimetric method (Paradee et al. 2019). In the assay, BRE, GTE, or standard gallic acid (GA) was incubated with a solution comprising 10% Folin–Ciocalteu reagent and 7.5% sodium carbonate, and left at room temperature for 30 min in the dark. After incubation, absorbance was measured at 760 nm using a microplate reader. TPC was calculated from the calibration curve (*y* = 0.0035x + 0.0663, *R* ^2^ = 0.9978) of GA and expressed as mg of gallic acid equivalent (GAE) per gram of extract.

### 2.7 Quantification of Total Flavonoid Content

Total flavonoid content (TFC) was assessed using an aluminum chloride colorimetric method (Paradee et al. 2019). Briefly, BRE, GTE, or standard quercetin (Q) was mixed with 10% aluminum chloride solution, 1 M potassium acetate, and deionized water for 30 min in the dark. Following this incubation, absorbance was measured at 415 nm using a microplate reader. TFC was determined based on a standard curve (*y* = 0.0051x + 0.1041, *R* ^2^ = 0.9910) of Q and expressed as mg of quercetin equivalent (QE) per g extract.

### 2.8 Quantification of Total Anthocyanin Content

Total anthocyanin content (TAC) was determined using the pH differential method, which is based on the structural changes of anthocyanins as the pH shifts between 1.0 and 4.5 (J. Lee et al. 2005). Briefly, BRE or GTE was incubated with two buffer systems: potassium chloride buffer (0.025 M, pH 1.0) and sodium acetate buffer (0.4 M, pH 4.5) for 30 min. Following incubation, the absorbance was measured at 520 nm and 700 nm using a spectrophotometer. The net absorbance (A) was calculated as: Absorbance (A) = (A_520_ − A_700_)_pH 1.0_ − (A_520_ − A_700_)_pH 4.5_. TAC was then calculated using the following formula: TAC (mg/L) = (A × MW × DF × 1000)/(ε × l), where A is the net absorbance, MW is the molecular weight of cyanidin‐3‐glucoside (449.2 g/mol), ε is the molar extinction coefficient (26,900 L/mol/cm), DF is the dilution factor, and l is the path length of the cuvette (1 cm). TAC was expressed as mg of cyanidin‐3‐glucoside (C3G) equivalents per gram of extract (mg C3G/g extract).

### 2.9 DPPH Radical‐Scavenging Assay

DPPH radical‐scavenging assay was employed to evaluate the antioxidant capacity of BRE or GTE (Chuljerm et al. 2023). In this test, BRE, GTE, or standard Trolox was incubated with 167 μM DPPH radical solution for 30 min in the dark. Following incubation, absorbance (A) was measured at 520 nm using a microplate reader. The percentage of DPPH radical‐scavenging activity was determined using the formula: Scavenging activity (%) = (1 − A_sample_/A_control_) × 100. The results were expressed in terms of IC_50_ values.

### 2.10 ABTS Radical‐Scavenging Assay

ABTS radical‐scavenging assay was employed to assess the antioxidant activity of BRE or GTE (Prommaban et al. 2022). ABTS radical working solution was prepared by the reaction of 7 mM ABTS with 2.45 mM of potassium persulfate at room temperature for 16 h in the dark. This solution was then diluted with ethanol to achieve an absorbance of 0.700 ± 0.020 at 734 nm. In the assay, BRE, GTE, or Trolox was combined with the ABTS radical solution and allowed to incubate at room temperature for 5 min in the dark. After incubation, absorbance (A) was measured at 734 nm against the reagent blank using a spectrophotometer. The ABTS radical scavenging activity was calculated using the formula: Scavenging activity (%) = (1 − Asample/Acontrol) × 100. The results were presented in terms of IC_50_ values.

### 2.11 Ferric Reducing Antioxidant Power (FRAP) Assay

FRAP assay was employed to determine the antioxidant activity of BRE or GTE (Prommaban et al. 2022). The FRAP reagent was freshly prepared by mixing 300 mM acetate buffer (pH 3.6), 10 mM 2,4,6‐tripyridyl‐s‐triazine (TPTZ) in 40 mM HCl, and 20 mM FeCl_3_ in a ratio of 10:1:1. For the assay, BRE, GTE, or Trolox was added to the FRAP reagent, then incubated at 37°C for 5 min in the dark to allow the reaction to complete. Absorbance was then measured at 595 nm by using a microplate reader. The antioxidant capacity of the extracts was expressed as mg Trolox equivalents per gram of extract (mg Trolox/g extract).

### 2.12 Cell Culture

Human hepatocellular carcinoma (HepG2) cells were cultured in Dulbecco's Modified Eagle Medium (DMEM) supplemented with 10% FBS and 100 IU/mL penicillin and streptomycin. HepG2 cells were maintained in a humidified 5% CO_2_ incubator at 37°C. The culture medium was refreshed every 2 days until the cells reached 70%–80% confluency, at which point they were subcultured.

### 2.13 Cell Viability Assay

Cell viability of HepG2 cells was determined using the MTT assay (Paradee et al. 2019). HepG2 cells were plated at a density of 1 × 10^6^4^ cells per well in a 96‐well plate and incubated for 24 h. The cells were then treated with various concentrations of oleic acids (OA), BRE, GTE, and the combination of BRE and GTE in a 3:1 ratio for 24 h. After incubation, MTT dye (5 mg/mL) was added to each well and incubated for an additional 4 h. The culture medium was subsequently removed and replaced with dimethyl sulfoxide (DMSO). Absorbance was measured at 570 and 630 nm using a microplate reader. The results were presented as the percentage of cell viability.

### 2.14 Quantification of Lipid Using AdipoRed Assay

Lipid accumulation in OA‐induced HepG2 cells was measured using AdipoRed Adipogenesis Assay Reagent (Tie et al. 2021). HepG2 cells were seeded in a 24‐well plate and incubated for 24 h. Lipid accumulation was then induced by treating the cells with OA for 24 h. Following induction, the HepG2 cells were treated with various concentrations of BRE, GTE, and the combination of BRE and GTE in a 3:1 ratio for an additional 24 h. After incubation, the cells were washed with PBS and stained with AdipoRed fluorescent dye for 10 min. Fluorescence intensity was measured using a fluorescent plate reader at an excitation wavelength of 485 nm and an emission wavelength of 572 nm. The cells were then photographed using an inverted fluorescence microscope.

### 2.15 Measurement of AST

AST levels in OA‐induced HepG2 cells were measured using an automated analyzer. Briefly, HepG2 cells were stimulated with OA for 24 h and then treated with various concentrations of BRE, GTE, and the combination of BRE and GTE in a 3:1 ratio for an additional 24 h. Following treatment, the culture medium was collected and then quantified for AST levels using an automated analyzer according to the manufacturer's instructions.

### 2.16 Measurement of Interleukin 6 (IL‐6)

IL‐6 levels in OA‐induced HepG2 cells were measured using a commercial ELISA kit. In this procedure, HepG2 cells were stimulated with OA for 24 h and then treated with various concentrations of BRE, GTE, and the combination of BRE and GTE in a 3:1 ratio for an additional 24 h. Following treatment, the culture medium was collected and centrifuged at 1500 rpm for 10 min at 4°C. The supernatant was then quantified for IL‐6 levels using a commercial ELISA kit according to the manufacturer's instructions.

### 2.17 Statistical Analysis

Data were expressed as mean ± standard deviation (SD). Statistical significance was determined using one‐way analysis of variance (ANOVA) followed by Tukey–Kramer post hoc test. *p* < 0.05 was considered a significant difference.

## 3 Results

### 3.1 Characterization of Anthocyanins, Catechins, and Tocopherols

As illustrated in Table 1, HPLC analysis identified both anthocyanins and catechins in BRE and GTE. BRE was found to have high levels of anthocyanins, particularly cyanidin‐3‐O‐glucoside chloride (C3G), cyanidin‐3‐O‐rutinoside chloride, and peonidin‐3‐O‐glucoside chloride (P3G), with concentrations of 48.55 ± 1.57, 0.52 ± 0.06, and 0.88 ± 0.02 mg/100 g extract, respectively. In contrast, BRE contained lower levels of catechins, including caffeine, GA, catechin, and epicatechin, with concentrations of 0.45 ± 0.04, 0.64 ± 0.05, 0.55 ± 0.01, and 0.04 ± 0.01 mg/100 g extract, respectively. On the other hand, GTE was rich in catechins, especially EGCG at 841.39 ± 26.31 mg/100 g extract. GTE also contained caffeine, GA, catechin (C), epicatechin (EC), and epicatechin gallate (ECG) at 134.49 ± 28.29, 1.73 ± 0.23, 141.37 ± 2.45, 4.42 ± 0.01, and 61.45 ± 2.69 mg/100 g extract, respectively. Additionally, GTE contained low levels of cyanidin‐3,5‐diglucoside (0.05 ± 0.01 mg/100 g extract). These findings highlight that BRE is predominantly composed of anthocyanins, especially C3G, whereas GTE is predominantly rich in catechins.

**TABLE 1:** HPLC analysis of anthocyanin, catechin and tocopherol contents in BRE and GTE.

Assigned compound	BRE (mg/100 g)	GTE (mg/100 g)
*Anthocyanin*		
Cyanidin‐3,5‐diglucoside (Cyanin chloride)	ND	0.05 ± 0.01
Cyanidin‐3‐O‐glucoside chloride (C3G)	48.55 ± 1.57	ND
Cyanidin‐3‐O‐rutinoside chloride	0.52 ± 0.06	ND
Peonidin‐3‐O‐glucoside chloride (P3G)	0.88 ± 0.02	ND
Caffeine	0.45 ± 0.04	134.49 ± 28.29
Gallic acid	0.64 ± 0.05	1.73 ± 0.23
*Catechin*		
Catechin (C)	0.55 ± 0.01	141.37 ± 2.45
Epicatechin (EC)	0.04 ± 0.01	4.42 ± 0.01
Epicatechin gallate (ECG)	ND	61.45 ± 2.69
EGCG	ND	841.39 ± 26.31
*Tocopherols*		
α‐Tocopherol	0.04 ± 0.01	0.41 ± 0.01
β‐Tocopherol and γ‐Tocopherol	3.38 ± 0.04	29.70 ± 0.11
δ‐Tocopherol	ND	143.53 ± 0.55

*Note:* Data are expressed as mean ± SD.

In addition to anthocyanins and catechins, tocopherols in BRE and GTE were also analyzed using HPLC. BRE contained alpha‐tocopherol of 0.04 ± 0.01 mg/100 g extract, and beta‐ and gamma‐tocopherols of 3.38 ± 0.04 mg/100 g extract. In contrast, GTE contained higher levels of tocopherols, with alpha‐tocopherol of 0.41 ± 0.01 mg/100 g extract, beta‐ and gamma‐tocopherols of 29.70 ± 0.11 mg/100 g extract, and delta‐tocopherol of 143.53 ± 0.55 mg/100 g extract. These findings indicate that GTE had a higher tocopherol content compared to BRE.

### 3.2 Total Phenolic, Flavonoid, and Anthocyanin Content of BRE and GTE

Table 2 shows the total phenolic, flavonoid, and anthocyanin contents of BRE and GTE. GTE had higher phenolic and flavonoid contents, with 520.2 ± 0.5 mg GAE/g extract and 11.6 ± 1.1 mg QE/g extract, respectively, compared to BRE, which had 111.2 ± 8.8 mg GAE/g extract and 5.6 ± 1.8 mg QE/g extract. In contrast, BRE had a higher anthocyanin content (18.61 ± 1.62 mg C3G/g extract) than GTE (0.20 ± 0.05 mg C3G/g extract). These findings indicate that BRE is rich in anthocyanins, while GTE is rich in phenolics and flavonoids.

**TABLE 2:** Total phenolic content (TPC), total flavonoid content (TFC), total anthocyanin content (TAC), and anti‐oxidant properties of BRE and GTE.

Extract/positive control	TPC (mg GAE/g extract)	TFC (mg QE/g extract)	TAC (mg C3G/g extract)	DPPH assay IC_50_ (μg/mL)	ABTS assay IC_50_ (μg/mL)	FRAP assay (mg Trolox/g extract)
BRE	111.2 ± 8.8	5.6 ± 1.8	18.61 ± 1.62	351.5 ± 36.5	264.3 ± 11.8	128.2 ± 4.4
GTE	520.2 ± 0.5	11.6 ± 1.1	0.20 ± 0.05	85.1 ± 22.0	79.0 ± 4.1	323.8 ± 6.8
Trolox	—	—	—	44.6 ± 3.7	41.0 ± 2.6	—

*Note:* Data from three independent experiments are expressed as mean ± SD.

### 3.3 Antioxidant Properties of BRE and GTE

**FIGURE 1:**
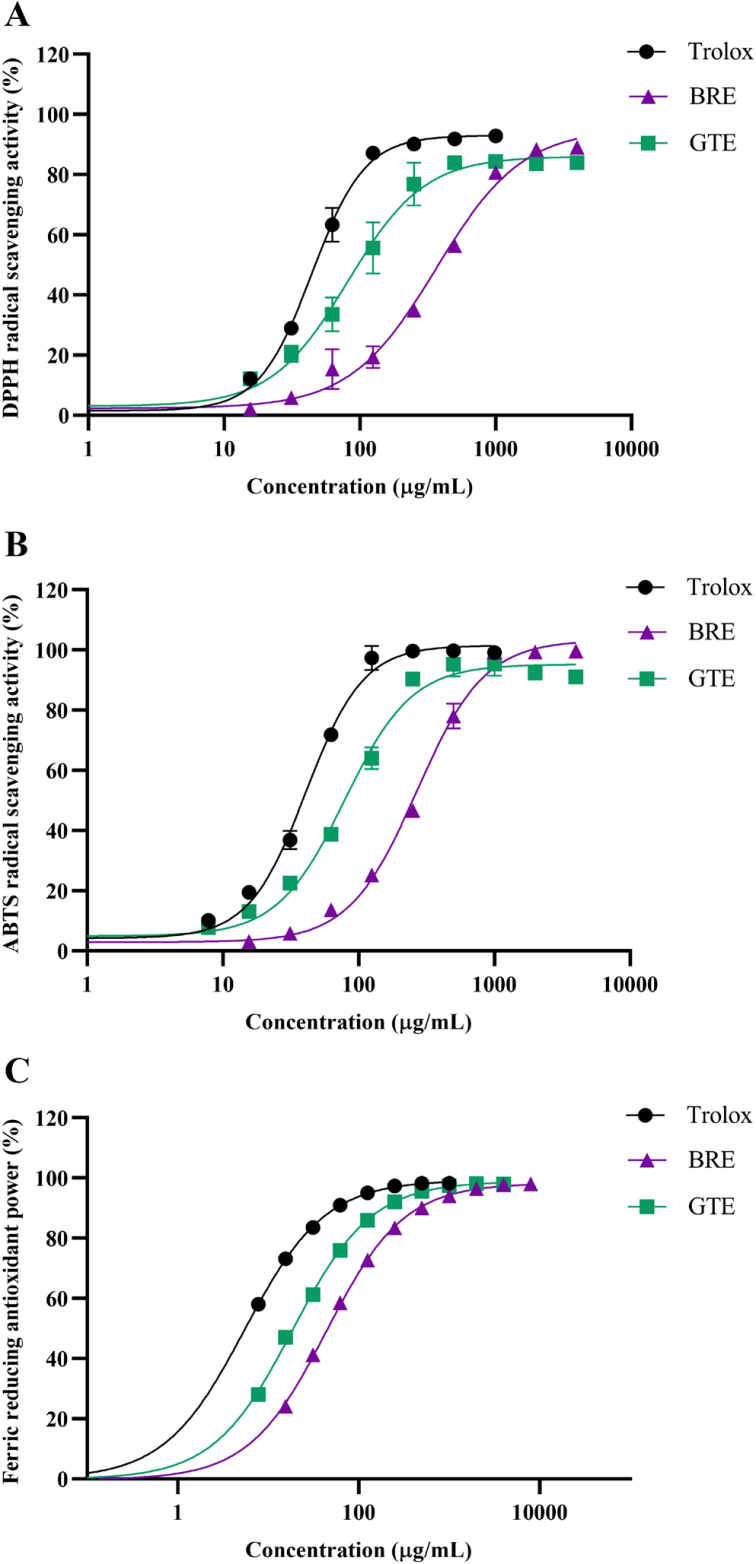
Antioxidant activities of BRE and GTE: (A) DPPH assay, (B) ABTS assay, and (C) FRAP assay. Data from three independent experiments are expressed as mean ± SD.

As shown in Table 2 and Figure 1, antioxidant properties of BRE and GTE were assessed using DPPH, ABTS radical scavenging assay, and FRAP assay. In the DPPH assay, Trolox, BRE, and GTE effectively scavenged the DPPH radical in a dose‐dependent manner (Figure 1A). Trolox, as a standard antioxidant, exhibited the highest DPPH scavenging efficacy with an IC_50_ of 44.6 ± 3.7 μg/mL, followed by GTE and BRE with IC_50_ values of 85.1 ± 22.0 and 351.5 ± 36.5 μg/mL, respectively.

In the ABTS assay, Trolox and both extracts effectively inhibited ABTS radicals in a concentration‐dependent manner (Figure 1B). Trolox presented the greatest ABTS radical scavenging activity with IC_50_ of 41.0 ± 2.6 μg/mL, followed by GTE and BRE with IC_50_ values of 79.0 ± 4.1 and 264.3 ± 11.8 μg/mL, respectively.

Similarly, in the FRAP assay, Trolox, BRE, and GTE demonstrated the ability to reduce ferric (Fe^3+^) to ferrous (Fe^2+^) ions in a concentration‐dependent manner (Figure 1C). GTE showed higher reducing power, with 323.8 ± 6.8 mg Trolox equivalents/g extract, compared to BRE, which had 128.2 ± 4.4 mg Trolox equivalents/g extract. These results suggest that GTE, which contains higher total phenolic and flavonoid content, possesses more potent antioxidant properties than BRE.

### 3.4 Cytotoxicity of OA, BRE, and GTE

**FIGURE 2:**
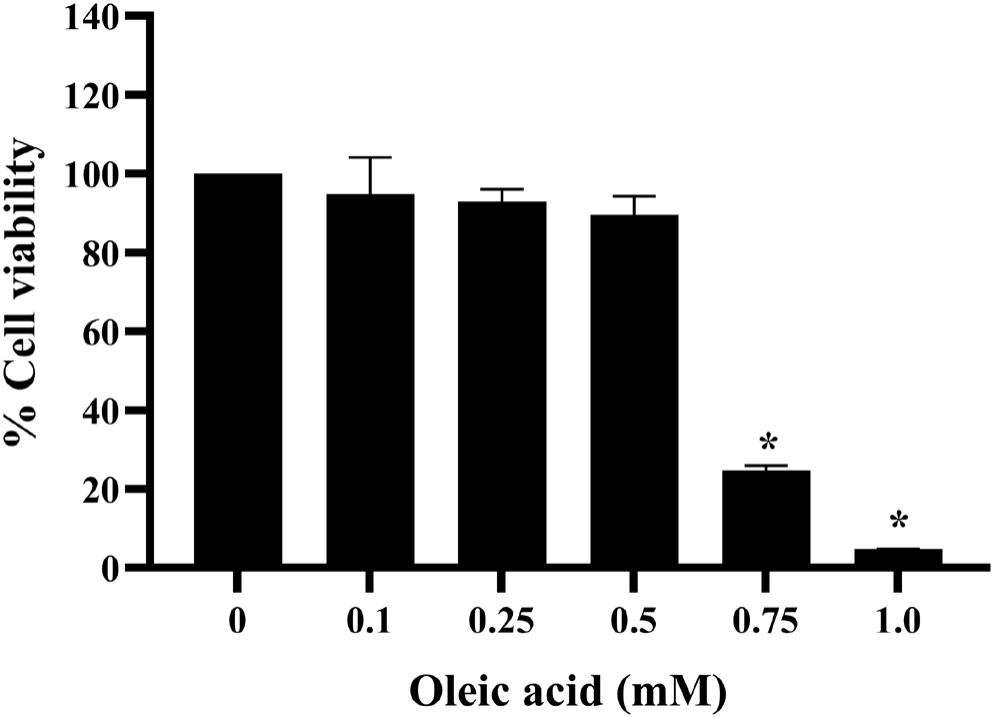
Cell viability of HepG2 cells after treatment with various concentrations of oleic acid for 24 h. Data from three independent experiments are expressed as mean ± SD. *p < 0.05 compared to the untreated control.

Cytotoxicity of OA in HepG2 cells was assessed using the MTT assay, as shown in Figure 2. After 24 h, OA at concentrations of 0.1–0.5 mM did not affect the cell viability of HepG2 cells compared to the untreated control. However, OA at concentrations of 0.75 and 1.0 mM reduced cell viability to less than 30%. These findings suggest that OA at concentrations below 0.5 mM is safe for HepG2 cells and is suitable for further testing.

**FIGURE 3:**
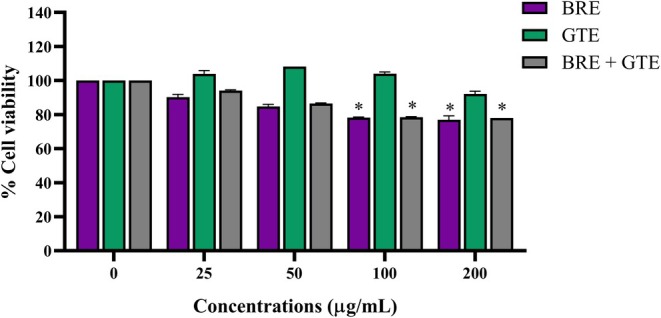
Cell viability of HepG2 cells after treatment with various concentrations of BRE, GTE, and the combination of BRE and GTE for 24 h. Data from three independent experiments are expressed as mean ± SD. *p < 0.05 compared to the untreated control.

The cytotoxicity of BRE, GTE, or the combination of BRE and GTE was also evaluated in HepG2 cells for 24 h. As illustrated in Figure 3, GTE at concentrations ranging from 25 to 200 μg/mL exhibited no toxicity to HepG2 cells after 24 h, with cell viability remaining above 80%. BRE and the combination of BRE and GTE at concentrations of 25–50 μg/mL maintained cell viability above 80% when compared to the untreated control. However, BRE and the combination of BRE and GTE at concentrations of 100–200 μg/mLsignificantly decreased cell viability below 80%. These results indicate that GTE, BRE, and the combination of BRE and GTE at concentrations below 50 μg/mL are non‐toxic to HepG2 cells and can be used for further testing.

### 3.5 Anti‐Lipid Accumulation of BRE, GTE, and the Combination of BRE and GTE

**FIGURE 4:**
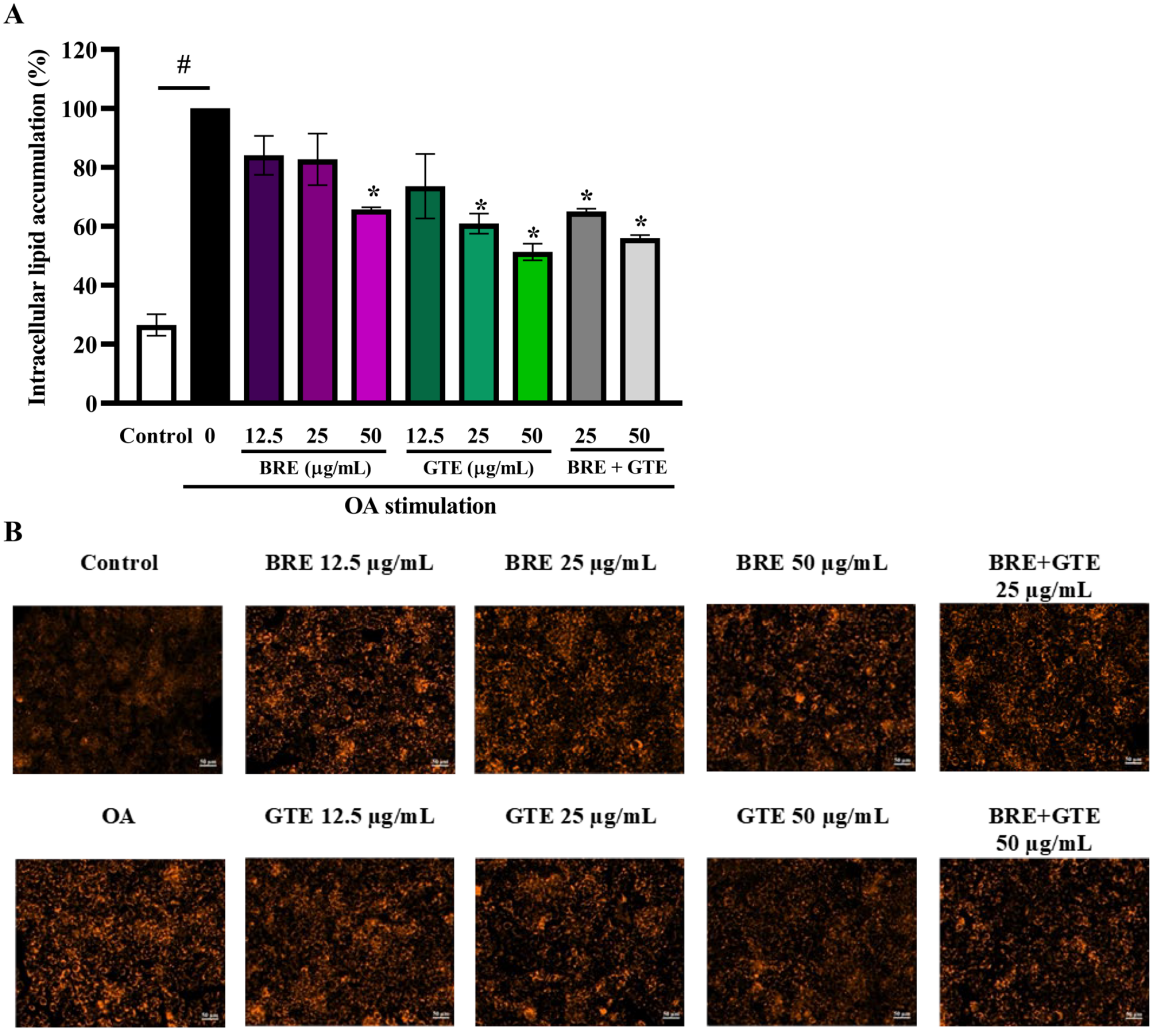
Lipid accumulation in oleic acid–induced HepG2 cells after treatment with various concentrations of BRE, GTE, and their combination for 24 h. (A) Levels of lipid accumulation and (B) images of intracellular lipid. Data from three independent experiments are expressed as mean ± SD. *p < 0.05 compared to the untreated control. ^#^ *p* < 0.05 compared to control.

The effects of BRE, GTE, and the combination of BRE and GTE on lipid accumulation in OA‐induced HepG2 cells are demonstrated in Figure 4A,B. After 24 h, 0.5 mM OA significantly increased lipid accumulation in HepG2 cells compared to the untreated control group. Treatment with BRE at concentrations of 12.5–50 μg/mL reduced lipid accumulation in a concentration‐dependent manner, with 50 μg/mL showing a significant effect. Similarly, GTE treatment at concentrations of 12.5–50 μg/mL effectively decreased the level of lipid accumulation in a dose‐dependent manner, with 25–50 μg/mL showing a significant effect. Moreover, the combination of BRE and GTE at a dose of 25–50 μg/mL significantly inhibited lipid accumulation. The combination of BRE and GTE in a 3:1 ratio at a total concentration of 50 μg/mL, consisting of 37.5 μg/mL of BRE and 12.5 μg/mL of GTE, showed a trend toward reducing lipid accumulation more effectively than BRE alone at 50 μg/mL and GTE alone at 12.5 μg/mL. These findings are consistent with the lipid accumulation observed in the cellular images shown in Figure 4B, suggesting that both BRE, GTE, and their combination possess the ability to reduce lipid accumulation in OA‐induced liver cells.

### 3.6 Anti‐Liver Damage Effects of BRE, GTE, and the Combination of BRE and GTE

**FIGURE 5:**
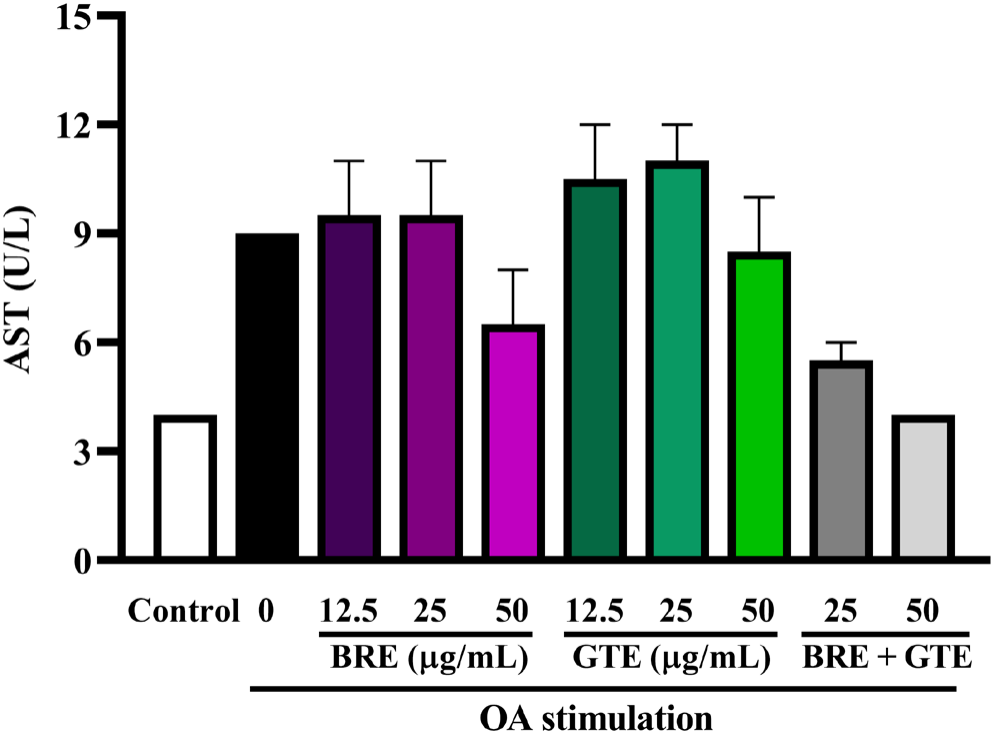
AST levels in oleic acid‐induced HepG2 cells after treatment with various concentrations of BRE, GTE, and their combination for 24 h. Data from three independent experiments are expressed as mean ± SD. *p < 0.05 compared to the untreated control.

The effects of BRE and GTE on liver enzyme activity in OA‐induced HepG2 cells are shown in Figure 5. Treatment with 0.5 mM OA for 24 h elevated AST levels compared to the untreated control group, indicating liver cell damage. Treatment with BRE or GTE at a concentration of 50 μg/mL decreased AST activity compared to the OA‐treated group. The combination of BRE and GTE at a dose of 25–50 μg/mL attenuated the AST activity to levels observed in the untreated control group. These results suggest that BRE, GTE, and their combination exhibit hepatoprotective effects.

### 3.7 Anti‐Inflammatory Effects of BRE, GTE, and the Combination of BRE and GTE

**FIGURE 6:**
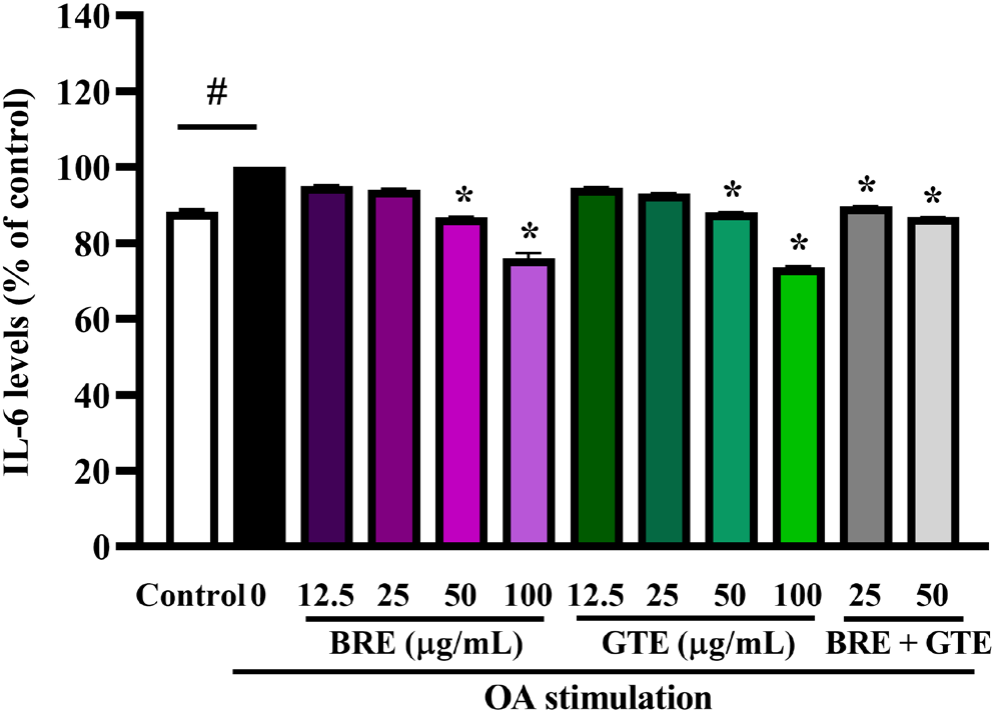
IL‐6 levels in oleic acid‐induced HepG2 cells after treatment with various concentrations of BRE, GTE, and their combination for 24 h. Data from three independent experiments are expressed as mean ± SD. *p < 0.05 compared to the untreated control. ^#^ *p* < 0.05 compared to control.

As shown in Figure 6, the anti‐inflammatory effects of BRE, GTE, and the combination of BRE and GTE were evaluated by measuring IL‐6 levels in OA‐induced HepG2 cells. After 24 h, OA significantly increased IL‐6 secretion compared to the untreated control group, indicating hepatic inflammation. Treatment with BRE or GTE at concentrations of 12.5–100 μg/mL attenuated IL‐6 levels in a dose‐dependent manner, with 50–100 μg/mL demonstrating significant effects. The combination of BRE and GTE at a dose of 25–50 significantly decreased IL‐6 levels. The combination of BRE and GTE in a 3:1 ratio at a total dose of 50 μg/mL, consisting of 37.5 μg/mL of BRE and 12.5 μg/mL of GTE, showed a trend toward greater inhibition compared to GTE alone at 12.5 μg/mL. These findings suggest that both BRE and GTE, and their combination possess anti‐inflammatory properties in liver cells.

## 4 Discussion

This study highlights the potential of BRE, GTE, and their combination as therapeutic agents for managing hepatic steatosis. BRE was found to be rich in anthocyanins, particularly Cyanidin‐3‐O‐glucoside (C3G), consistent with previous studies showing C3G as the predominant anthocyanin (90%) in anthocyanin‐rich BRE (Li et al. 2023; Shozib et al. 2021). Similarly, GTE is rich in catechins, particularly EGCG, caffeine, catechin (C), epicatechin (EC), and epicatechin gallate (ECG). These findings agree with earlier reports identifying seven main catechins in green tea: (−)‐gallocatechin (GC), (−)‐epigallocatechin (EGC), (+)‐catechin (C), (−)‐epigallocatechin‐3‐gallate (EGCG), (−)‐epicatechin (EC), (−)‐gallocatechingallate (GCG), and (−)‐epicatechingallate (ECG) (Bonoli et al. 2003; Wangkarn et al. 2021). However, some catechins were not detected in our GTE, likely due to variations in solvent, temperature, and extraction methods of the extracts.

In the present study, GTE exhibited higher TPC and TFC compared to BRE, while BRE demonstrated a greater TAC than GTE. Previous studies reported that Italian green tea contained TPC of 149.18 mg GAE/g dry weight, while matcha green tea in Korea had TPC of 289.41 mg GAE/g extract and TFC of 100.57 mg of rutin equivalent (RE)/g (Falla et al. 2021; Kim et al. 2020). Although consistent with our findings, our GTE showed a higher TPC (520.2 mg GAE/g extract) than those reported in earlier studies. Additionally, another study found that ethanol extract of black rice contained TPC of 5.03 mg GAE/g extract, TFC at 4.79 mg catechin equivalent (CE)/g extract, and TAC of 1.09 mg C3G/g extract (Tyagi et al. 2022). Our results are in accordance with these findings, as BRE had TPC of 111.2 mg GAE/g extract, TFC of 5.6 mg QE/g extract, and TAC of 18.61 mg C3G/g extract, but with higher values than those reported previously. These variations in phenolic, flavonoid, and anthocyanin content likely depend on differences in plant cultivars, solvents, and extraction methods.

Antioxidant results demonstrated that GTE, which contained higher phenolic and flavonoid content, exhibited superior DPPH and ABTS radical scavenging properties, as well as a more effective reduction of ferric to ferrous ions, compared to BRE. Our findings are consistent with earlier reports, which showed that GTE had potent antioxidant capacities, with 1423.22, 4293.33, and 1555.06 mM Trolox equivalent/g extract for the DPPH, ABTS, and FRAP assays, respectively. It was found that EGCG, with the highest number of phenolic hydroxyl groups, had the highest antioxidant activities in DPPH, ABTS, and FRAP assays, followed by GCG, ECG, EGC, GC, EC, and C (L. S. Lee et al. 2014). Furthermore, our findings are consistent with previous literature, which reported that black rice ethanolic extract effectively scavenged DPPH and ABTS radicals with IC_50_ values of 109.61 and 127.74 μg/mL, respectively (Tyagi et al. 2022). Additionally, BRE demonstrated reducing power in the FRAP assay with an IC_50_ of 97.59 μg/mL. Another study found that ethanolic extracts of red rice and black rice had IC_50_ values of 43.43 and 23.92 μg/mL, respectively, for DPPH radical scavenging (Sukrasno et al. 2017). Although the IC_50_ values for BRE reported in previous studies were lower than those observed in our study, this variation may be attributed to differences in the phenolic, flavonoid, and anthocyanin content of the extracts. It is likely that phenolic, flavonoid, and anthocyanin contribute significantly to the antioxidant properties of plant extracts.

Excessive fat intake can result in an imbalance between lipid storage and fat export in the liver, leading to fat accumulation and the development of steatosis. In this study, we evaluated the effects of BRE, GTE, as well as their combination on lipid accumulation, inflammatory cytokines, and liver damage (as measured by AST activity) in OA‐induced HepG2 cells. OA which is an unsaturated fatty acid, significantly increased lipid accumulation, oxidative stress, and inflammation in HepG2 cells, simulating the progression of hepatic steatosis. Our results showed that BRE, GTE, and their combination significantly inhibited lipid accumulation, with GTE exhibiting more potent activity compared to BRE. This enhanced lipid‐lowering activity of GTE may be attributed to its higher content of phenolic compounds and catechins. Consistent with a previous study, Chinese green tea ameliorated fat accumulation in high‐fat diet (HFD)‐induced obese mice by suppressing fatty acid synthase (FAS) (C. Liu et al. 2019). Additionally, green tea has been reported to decrease pro‐inflammatory cytokines, such as IL‐6 and TNF‐α, in HFD‐induced mice (Chen et al. 2022). Similarly, BRE has been found to reduce hepatic cholesterol and triglyceride accumulation in HFD‐induced obese mice through the activation of fatty acid oxidation (Jang et al. 2012; D. Liu et al. 2020). Another study reported that black rice anthocyanin extract inhibited TNF‐α and IL‐6 levels in dextran sulfate sodium (DSS)‐induced inflammation in mice (Li et al. 2023). These findings are in accordance with our results, which demonstrate that both GTE and BRE reduce inflammation by suppressing IL‐6 levels, thereby improving liver damage. The observed hepatoprotective effects of BRE, GTE, and their combination may be attributed to their phenolic, flavonoid, and anthocyanin content, which likely contribute to the reduction of lipid accumulation, oxidative stress, and inflammation associated with hepatic steatosis.

## 5 Conclusions

This study demonstrates that BRE is rich in anthocyanins, particularly Cyanidin‐3‐O‐glucoside (C3G), while GTE is abundant in catechins, especially EGCG. GTE had higher TPC and TFC, whereas BRE had greater TAC. Indeed, GTE exhibited more effective antioxidant properties compared to BRE, including superior radical scavenging and ferric‐to‐ferrous ion reduction. Interestingly, BRE, GTE, and the combination of BRE and GTE significantly reduced lipid accumulation, liver damage, and inflammation in OA‐induced HepG2 cells. These findings suggest that black rice, green tea, and their combination would be served as therapeutic agents for managing hepatic steatosis. Further research is warranted to elucidate the underlying mechanisms and to study these effects in animal models.

## Author Contributions

**Narisara Paradee:** investigation (equal), methodology (equal), visualization (equal), writing – original draft (equal). **Thararat Yimcharoen:** investigation (equal). **Niramon Utama‐ang:** conceptualization (equal), funding acquisition (equal), supervision (equal). **Kornvipa Settakorn:** investigation (equal). **Hataichanok Chuljerm:** investigation (equal). **Somdet Srichairatanakool:** conceptualization (equal), funding acquisition (equal), supervision (equal). **Pimpisid Koonyosying:** conceptualization (equal), methodology (equal), project administration (equal), visualization (equal), writing – review and editing (equal).

## Conflicts of Interest

The authors declare no conflicts of interest.

## Data Availability

The data that support the findings of this study are available from the corresponding author upon reasonable request.
